# Proton Therapy in Non-Rhabdomyosarcoma Soft Tissue Sarcomas of Children and Adolescents

**DOI:** 10.3390/cancers16091694

**Published:** 2024-04-26

**Authors:** Sabina Vennarini, Francesca Colombo, Alfredo Mirandola, Ester Orlandi, Emilia Pecori, Stefano Chiaravalli, Maura Massimino, Michela Casanova, Andrea Ferrari

**Affiliations:** 1 Pediatric Radiotherapy Unit, Fondazione IRCCS Istituto Nazionale Tumori, 20133 Milan, Italy; sabina.vennarini@istitutotumori.mi.it (S.V.); emilia.pecori@istitutotumori.mi.it (E.P.); 2 Radiation Oncology Unit, Clinical Department, CNAO National Center for Oncological Hadrontherapy, 27100 Pavia, Italy; francesca.colombo@cnao.it (F.C.); ester.orlandi@cnao.it (E.O.); 3 Medical Physics Unit, Clinical Department, CNAO National Center for Oncological Hadrontherapy, 27100 Pavia, Italy; alfredo.mirandola@cnao.it; 4 Department of Clinical, Surgical, Diagnostic, and Pediatric Sciences, University of Pavia, 27100 Pavia, Italy; 5 Pediatric Oncology Unit, Fondazione IRCCS Istituto Nazionale Tumori, 20133 Milano, Italy; stefano.chiaravalli@istitutotumori.mi.it (S.C.); maura.massimino@istitutotumori.mi.it (M.M.); michela.casanova@istitutotumori.mi.it (M.C.)

**Keywords:** non-rhabdomyosarcoma soft tissue sarcomas, NRSTS, children, adolescents, proton beam therapy, local treatment, radiotherapy

## Abstract

**Simple Summary:**

Proton Beam Therapy (PBT) is an interesting therapeutic option for children and adolescents with non-rhabdomyosarcoma soft tissue sarcomas (NRSTS). In fact, if it is true that radiotherapy is a key part of the multi-modal treatment of NRSTS patients, it is also true that the risk of radiation-induced side effects represent an important limitation in its use. The unique characteristics of protons can be leveraged to minimize doses to healthy tissue, potentially allowing for increased tumor doses and enhanced preservation of surrounding tissues. International cooperative efforts are required to better define the indications for PBT (based on the patient’s age, estimated outcome, and tumor location), taking into account the currently limited number of available proton therapy facilities.

**Abstract:**

This paper provides insights into the use of Proton Beam Therapy (PBT) in pediatric patients with non-rhabdomyosarcoma soft tissue sarcomas (NRSTS). NRSTS are a heterogeneous group of rare and aggressive mesenchymal extraskeletal tumors, presenting complex and challenging clinical management scenarios. The overall survival rate for patients with NRSTS is around 70%, but the outcome is strictly related to the presence of various variables, such as the histological subtype, grade of malignancy and tumor stage at diagnosis. Multimodal therapy is typically considered the preferred treatment for high-grade NRSTS. Radiotherapy plays a key role in the treatment of children and adolescents with NRSTS. However, the potential for radiation-induced side effects partially limits its use. Therefore, PBT represents a very suitable therapeutic option for these patients. The unique depth-dose characteristics of protons can be leveraged to minimize doses to healthy tissue significantly, potentially allowing for increased tumor doses and enhanced preservation of surrounding tissues. These benefits suggest that PBT may improve local control while reducing toxicity and improving quality of life. While clear evidence of therapeutic superiority of PBT over other modern photon techniques in NRSTS is still lacking—partly due to the limited data available—PBT can be an excellent treatment option for young patients with these tumors. A dedicated international comprehensive collaborative approach is essential to better define its role within the multidisciplinary management of NRSTS. Shared guidelines for PBT indications—based on the patient’s age, estimated outcome, and tumor location—and centralization in high-level referral centers are needed to optimize the use of resources, since access to PBT remains a challenge due to the limited number of available proton therapy facilities.

## 1. Introduction

“Non-rhabdomyosarcoma soft tissue sarcomas” (NRSTS) is the term generally used in pediatric oncology to describe the very heterogeneous group of mesenchymal extraskeletal malignant tumors different from rhabdomyosarcoma [1,2]. This group includes over 50 distinct histological subtypes that can manifest anywhere in the body. These subtypes are classified on a histological basis according to the specific adult tissue they resemble. NRSTS histotypes may have different biology and clinical behaviors varying from relatively benign to highly aggressive [3]. The rarity and the heterogeneity of NRSTS make their management complex and challenging, and suggest that children and adolescents with these tumors should be referred to selected experienced centers with multidisciplinary skills and the ability to enroll patients in clinical trials [4].

Overall, the cure rate for NRSTS patients is around 70% [5,6,7]. However, survival strictly depends on the presence of a number of variables, such as the histotype and grade of malignancy, disease extension/stage at diagnosis (including the degree of the initial surgery and tumor size), and tumor site [5,6,7,8,9,10]. These factors, which are significant in pediatric cases, also play a prognostic role in adults [11,12,13,14,15,16,17]. Notably, treatment outcomes for many NRSTS subtypes reported in pediatric cases are more favorable than those reported in adult cohorts [2].

Surgery remains the mainstay of treatment for most NRSTS. However, for high-grade tumors, multimodal therapy, including radiotherapy and chemotherapy, is frequently considered the most effective approach [3].

Like their adult counterparts, pediatric NRSTS are generally assumed to be relatively less responsive to chemotherapy than pediatric-type sarcomas such as rhabdomyosarcoma or Ewing sarcoma. Though NRSTS subtypes vary not only in their biology and clinical behavior, but also in their sensitivity to therapy, tumor response is reported in the range of 40–50% or less in unresected NRSTS series [18]. However, standard systemic therapy (i.e., ifosfamide–doxorubicin chemotherapy) may be specifically indicated for (1) patients with metastatic disease [19,20]; (2) as neo-adjuvant treatment in patients with locally advanced disease, to reduce tumor size and make such cases amenable to conservative complete resection as well as to promptly treat any micro-metastases [18,21,22,23]; and (3) as adjuvant chemotherapy in high-grade and large tumors, to prevent distant recurrences after initial surgery [5,6,14,15,16,17,24].

Radiotherapy (RT) plays a key role in the treatment of NRSTS. RT—and its doses and volumes—may be indicated selectively based upon factors such as resectability and margins status; histological type and grade; tumor stage, size, and anatomical site; and chemo-responsiveness [25]. However, also the age of patients should be taken into account as a major variable. The indications for RT, in fact, may be more limited in young children due to the higher risk of severe late effects, and should be customized with the aim of achieving local control while taking into account the risk of radiation-induced sequelae and the preservation of function [3].

RT can be used either in neoadjuvant or adjuvant settings. In initially resected NRSTS, post-operative RT has a role in local control after incomplete resections and after wide excisions in the case of large and high-grade tumors [26,27,28]. Conversely, RT can be safely omitted in completely resected low-grade NRSTS and possibly in low-grade tumors after initial R1 resection (microscopically positive margins) [5,6]. The omission of RT has also been suggested in specific cases, including high-grade tumors (such as synovial sarcoma) less than 5 cm, after initial R0 surgery [29].

In patients with initially unresected NRSTS, the combination of RT and delayed surgery may vary according to the diverse clinical situations encountered, considering the age of patients, as well as the histotype, the tumor dimension, the tumor location, and the resectability of the disease. This strategy aims for the “best possible local treatment” to maximize local control chances while minimizing sequelae and preserving function. Treatment options include surgery alone, a combination of surgery and RT, or definitive RT when surgery would result in unacceptable morbidity [6,24,30,31].

Neo-adjuvant pre-operative RT has been increasingly used. In combination with chemotherapy, it may result in a high likelihood of surgical R0 margins at delayed resection. In addition, it may also help reduce both the dose and volume required (thus diminishing long-term morbidity) and improve effectiveness in non-hypoxic tissues [32,33,34,35].

**Figure 1:**
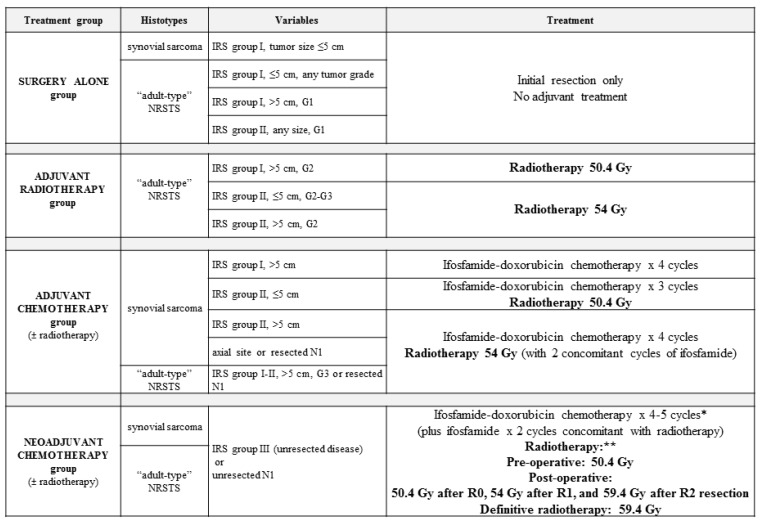
Risk-adapted treatment recommendations for localized adult-type NRSTS (including synovial sarcoma), according to the European pediatric Soft tissue sarcoma Study Group (EpSSG). IRS = Intergroup Rhabdomyosarcoma Study; IRS group I, complete resection at first surgery (initial R0 surgery); IRS group II, microscopic residual disease (after initial R1 surgery); IRS group III, biopsy or initial macroscopic residual disease (after R2 surgery); G = tumor grade. N1 = nodal involvement. * 4 cycles for synovial sarcoma, 5 courses for the other adult-type NRSTS. ** The EpSSG recommends customizing the “best possible local treatment” with the aim of maximizing the chances of local control, while minimizing treatment sequelae (for example, post-operative radiotherapy might be avoided in young patients—less than 6 years old—while it should be recommended in case of large tumors or after poor response to neo-adjuvant chemotherapy).

In order to summarize the indication for RT, Figure 1 describes the different NRSTS treatment categories developed according to the risk stratification by the European pediatric Soft Tissue Sarcoma Study Group (EpSSG) [6]. These treatment recommendations are applied to localized adult-type NRSTS (including synovial sarcoma). The EpSSG NRSTS 2005 protocol, together with the ARST0332 study developed by the North-American Soft Tissue Sarcoma Committee of the Children’s Oncology Group (COG) [5], sets the benchmark for the clinical management of NRSTS, establishing the currently adopted risk-adapted standards of care.

## 2. Proton Beam Therapy

RT plays a key role in the treatment of children and adolescents with NRSTS, but its use is partly limited by the potential radiation-induced side effects. The technological advances towards more effective and less toxic treatments is a crucial step in pediatric oncology. In this scenario, Proton Beam Therapy (PBT) represents a very suitable therapeutic choice for these patients: this external RT technique has evolved considerably since its first applications in the early 1990s [36], and it must be currently considered a valid therapeutic option for many clinical situations [37].

PBT is the most widely used form of hadron therapy: protons—as well as other ions like helium, carbon, or oxygen—have particular physical and biological properties [38]. In particular, the inverse depth-dose profile of protons in comparison to photons, and the sharp distal fall-off after the Bragg peak permit to limit the dose to the organs at risk crossed by the beams [39]. Given these characteristics, a smaller number of beam ports can be used: this reduces the “dose-bath” given to patients, as for volumetric photon techniques. In addition, it is of note that proton energy deposition is 10% more effective than photons; thus, the usually adopted Relative Biological Effectiveness (RBE) is 1.1 (or even higher according to many radiobiological models) [40].

Currently, the most prevalent delivery technique is the active pencil beam scanning (PBS). This involves scanning a pencil beam in transversal directions (up to a 40 × 40 cm field size) using scanning magnets, while adjusting the beam depth along its direction by inserting absorbers along the beam path (for cyclotrons). In contrast, synchrotrons permit direct beam energy alterations without the necessity for beam absorbers. The typical proton energy range, in water, varies from 30 to 300 mm, thus enabling the treatment of numerous tumor sites.

Proton therapy is particularly indicated for childhood cancers [41]. The unique physical and radiobiological properties of proton beams, together with the increasing accuracy in irradiation volume definition enabled by the latest imaging techniques, present significant benefits that could be translated into improved clinical outcomes. One of the principal advantages of PBT is the sparing of healthy tissues, which allows for dose escalation and potentially leads to improved local control and survival outcome in radioresistant neoplasms [42]. The sparing of healthy tissues also reduces acute toxicities during multimodal treatment, improving therapeutic adherence in pediatric malignancies requiring concomitant chemotherapy [43].

Although the dosimetric advantages of protons over photons are evident, this radiation technique requires a specialized team of physicists and physicians capable of managing the inherent difficulties that this technique entails. This is especially true for NRSTS, which can lead to different locations that can spread anywhere in the body. The management of range uncertainties (potentially leading to suboptimal dose distributions and treatment plans) require particular attention. The potential shift of the proton sharp distal dose fall-off might lead to two severe consequences, i.e., an underestimation of dose to the target or an overdose to the organs at risk distal to the beam direction [44]. Range uncertainties stem from organ motion, setup and anatomical changes, dose calculation approximations, and biological variables. In order to account for both setup errors and range uncertainties, robust plan optimization is highly recommended when using protons [45]. Range uncertainties, RBE variation (even higher than 1.1 especially in the distal edge of the field specific delivered dose) and plan robustness must be carefully accounted for, especially when the tumor localization is critical due to the surrounding organs at risk or when the beams traverse emptying/filling cavities or moving targets or organs at risk. In such instances, assessing organ motion is necessary or at least strongly recommended. It is recommended that mitigation strategies, such as the breath hold technique (in which the beam is on during the phase in which the patient holds their normal breath) and respiratory gating (in which the beam is on in a specific and predetermined respiratory phase), be applied during both the planning phase and the treatment phase.

## 3. Proton Beam Therapy in Pediatric NRSTS

PBT is emerging as a treatment option for soft tissue sarcoma therapy in pediatric and adolescent patients. To evaluate the role of PBT in this specific patient group, we conducted a comprehensive analysis of the scientific literature. The bibliographic search was performed on the NCBI PubMed, Embase and Cochrane Library databases in December 2023. The search terms used were ‘pediatric’, adolescents, ‘proton’, ‘radiotherapy’, ‘soft tissue sarcoma’, and ‘NRSTS’. We then used also ‘synovial sarcoma’ and ‘malignant peripheral nerve sheath tumor (MPNST)’, which are the two most frequent NRSTS in children and adolescents. We found 10 articles describing series of pediatric or adolescent patients with NRSTS treated with PBT, focusing on survival outcomes and toxicity, as reported in Table 1.

Four of the articles were case reports that included rare clinical situations located in critical sites [46,47,48,49].

A major contribution is that from the Center for Proton Therapy, Paul Scherrer Institute, Villigen, Switzerland, reporting on a series of 36 patients diagnosed with peripheral nerve sheath tumor (5 cases) and MPNST (31 cases). The study demonstrated good tolerance to hadron therapy treatment, with only five cases of grade 3 acute toxicities and two cases of grade 3 late toxicities. The authors highlighted the relevance of dose sparing to organs at risk achieved with PBT, especially considering the frequent association of this pathology with neurofibromatosis type 1 (NF-1), which caused a high risk of radiation side effects, as well as the development of secondary tumors [50].

Other published studies focused on large case series of patients with head and neck neoplasms [51,52] or skull base neoplasms [53], among which a minority were NRSTS cases. The reported toxicity and outcome data mainly refer to the overall population analyzed. The study from the Paul Scherrer Institute on adolescents and young adults with head and neck tumors reported also data on quality of life of surviving patients. This is particularly important in the adolescent and young adult age group, where the disease and its treatments can lead to alterations in physical, psychological, social, and emotional development. Although the sample size is limited and statistical significance cannot be established, the analysis conducted by Vazquez et al. highlighted the adverse effects of the disease on the daily lives of surviving patients. The impact is mainly attributed (71.4%) to the sequelae of anti-tumor treatments and to a lesser extent (14.3%) to the persistence of neoplastic symptoms. Treatment sequelae also have a negative impact on social interactions [52].

A paper by Hwang et al. described the UK Proton Overseas Programme (POP), which was launched in 2008 to centralize data collection and outcome analysis for all National Health Service-funded UK patients referred and treated abroad with PBT. The study reports on 495 patients (93% younger than 25 years) with non-central nervous system neoplasms, including 37 NRSTS cases. The median PBT dose was 51 Gy and the local control for NRSTS was 84.4% [54].

Finally, it is worth mentioning the specific sub-analyses on radiotherapy of the COG ARST0332 trial. This study reported on 181 patients with non-metastatic NRSTS aged 2–30 years and showed that only six cases (3%) received PBT [28,55].

## 4. Lessons from Adult Sarcoma Experiences

It is important to note that dosimetric studies comparing photon and proton radiotherapy techniques for rare and highly heterogeneous neoplasms, both in location and histology, are unlikely to be found. Therefore, it is appropriate to refer to case series and guidelines related to soft tissue sarcomas in the adult population. PBT has become increasingly popular among experts in adult sarcoma treatment in recent years. The NCCN 2023 guidelines for soft tissue sarcomas also recommend the use of sophisticated treatment plans, including Intensity Modulated Radiotherapy (IMRT) and/or protons, when External Beam Radiotherapy (EBRT) is employed, to improve the therapeutic ratio [56]. This recommendation applies to locations such as the head–neck, extremities, chest wall, and retro-peritoneal/intra-abdominal areas.

Regarding the extremities, the most common site of soft tissue sarcoma presentation, we can refer to dosimetric comparative studies conducted in the adult population, although the sample sizes are limited. A Mayo Clinic analysis of 14 adult patients with soft tissue sarcoma in the extremities, who were treated with pre-operative PBT (PBS-PBT up to a total dose of 50 Gy in 25 fractions), showed a significant reduction in dose to surrounding soft tissue and bone. Additionally, the treatment plan exhibited a greater degree of conformation and homogeneity compared to photon techniques. This intervention may decrease the frequency of complications at the surgical scar site, which is the most common toxicity in the neoadjuvant setting. Additionally, it may reduce the occurrence of late toxicities such as bone fractures, lymphoedema, fibrosis, skin changes, and reduced musculoskeletal function and strength [57].

Furthermore, in the adjuvant setting, a previous study in 10 patients demonstrated an increase in proton plane homogeneity and a reduction in low dose. However, it did not provide any benefit in terms of dose sparing to adjacent bone tissue [58].

A dosimetric analysis was conducted on eight adult patients with retro-peritoneal and intra-abdominal sarcomas undergoing pre-operative RT, comparing 3DCRT (three-Dimensional Conformal Radiation Therapy), IMRT, and 3DCPT (three-Dimensional Conformal Proton Therapy) techniques. The results showed that, with equal coverage of the clinical target volume (CTV), PBT had a clear advantage in terms of dose sparing for the bowel, ipsilateral and contralateral kidney, and liver. The studies also observed a significant reduction in integral dose, which is correlated with the risk of developing radio-induced secondary neoplasms [59].

Although specific dosimetric studies on pediatric NRSTS are scarce, insights can be drawn from research on rhabdomyosarcomas, given the similarity in disease sites. Several dosimetric studies have confirmed that PBT is an excellent therapeutic option for pediatric patients with rhabdomyosarcoma in various primary disease sites, such as the head–neck, pelvic, and paravertebral regions. These studies have demonstrated the clear advantage of proton therapy in sparing healthy tissue surrounding the treatment volume, with good tolerability in terms of acute and late toxicity and survival outcomes that are comparable to previously reported series in the literature [60].

## 5. Discussion

The role of RT in managing patients with NRSTS is highly complex. The cost–benefit balance of RT in the context of the multidisciplinary management is challenging and often requires significant decision-making due to various clinical variables involved, including patient’s age. The importance of RT in local disease control should be always balanced with the risk of medium- to long-term morbidity and morpho-functional sequelae that could heavily affect the quality of life of survivors, in particular in young patients who are still growing [3].

NRSTS presents a challenge in clinical decision-making for the elective indication as well as for the best and most appropriate technological choice due to its heterogeneity in terms of patient age, tumor subtype and grade, size and stage, and primary site [25,28]. Notably, the evolution of the radiotherapeutic context in NRSTS also stems from the completion of a limited number of specific pediatric prospective clinical studies, and the reliance on recommendations for RT derived from clinical studies in adults. Despite limited clinical experiences described in the literature, external beam radiotherapy techniques using photons have significantly advanced also in the treatment of NRSTS. We have transitioned from the use of conventional 3D conformal radiotherapy (3DCRT) to more sophisticated techniques of dose conformality and modulation on large and challenging targets, utilizing intensity-modulated radiotherapy (IMRT) and even volumetric-modulated arc therapy (VMAT).

Although published data show improved local control with the evolution of photon techniques in the treatment of NRSTS [58,61], the widespread advent of particle therapy facilities worldwide is generating significant interest in the pediatric sarcoma community, aiming to identify effective and less toxic treatment.

While formal evidence of superiority of PBT over other radiotherapy modalities is still needed (in NRSTS as in other tumors), the increasingly precise definition of irradiation volumes supported by dedicated imaging and the physical and radiobiological characteristics of the proton beam represent advantages that can reduce the exposure of organs at risk near the tumor and the integral dose to healthy tissues.

The access of young patients with NRSTS (and more in general with solid tumors) to PBT remains, however, a challenge. For example, it is worth mentioning the North American study involving 12,101 pediatric patients with various solid neoplasms treated in the 2004–2013 period, which reported an 8% proportion of patients receiving PBT (the percentage was higher in recent years, in younger patients, and in patients with private care and higher household income) [62].

The limited number of available PBT facilities remains a major issue. For example, in Italy, only two proton therapy centers are currently active (with few others coming in the near future), and this strongly limits the number of patients that can receive PBT. In addition, it makes it difficult to gather clinical data to be compared with photon treatments (the published series or case reports on NRSTS patients undergoing PBT are limited in number and heterogeneous in terms of tumor subtypes, sites and age range).

**Figure 2:**
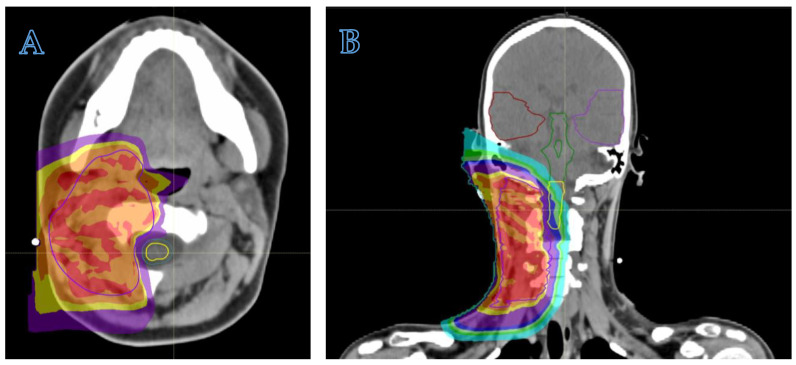
The axial (**A**) and coronal (**B**) views of a proton beam plan, utilizing a pencil scanning proton beam therapy (PBS-PBT) technique, illustrate the treatment of a 13-year-old girl with epithelioid sarcoma. The images demonstrate that the clinical target volume (CTV) is adequately covered by the high doses of radiotherapy (isodoses of 100%, 98% and 95% of the prescribed dose, respectively, indicated in red, orange and yellow). Meanwhile, the low doses of radiotherapy (isodoses of 60%, 50% and 25% in light green, green and blue, respectively) spare the organs at risk located in the supra- and subtentorial regions and the contralateral laterocervical region.

**Figure 3:**
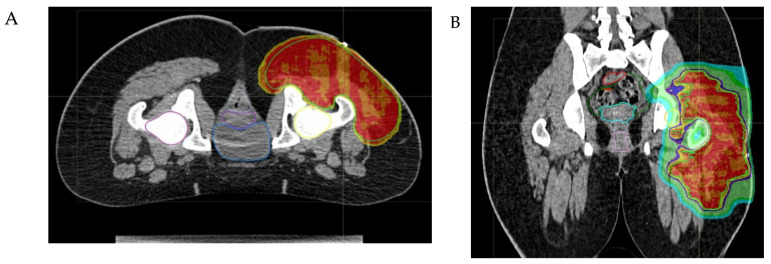
The axial (**A**) and coronal (**B**) views of a proton beam plan illustrate the treatment of a malignant peripheral nerve sheath tumor (MPNST) in a 15-year-old girl, using a pencil-scanning proton beam therapy (PBS-PBT) technique. The images demonstrate the optimal coverage of the clinical target volume (CTV) by the high radiotherapy doses (isodoses of 100%, 98% and 95% of the prescribed dose, respectively indicated in red, orange and yellow), and the total sparing of the abdominal–pelvic risk organs, including the reproductive organs.

Therefore, it is important to define shared indications for patients with NRSTS. PBT should be recommended according to different variables such as patient’s age, estimated outcome, and tumor site:

- PBT should be recommended for young children (for example, younger than 3 or 6 years of age) in order to minimize exposure to medium-to-low radiation doses and the risk of long-term side effects.
- Since the limited availability of PBT, this technique should be recommended to patients with relatively good prognosis (therefore, it should not be indicated in metastatic patients).
- The anatomical site and the subsequent fragility of the surrounding organs at risk to potential radiation damage is a critical matter. The head–neck, craniofacial, intra-abdominal, pelvic, and paravertebral regions may be considered elective sites, where PBT can minimize radiation exposure to nearby organs (Figure 2). In addition to minimizing the risk of late sequelae, the reduced irradiation of surrounding tissues (mucosae, for example) may also reduce acute toxicity and thus improve compliance with intensive multimodal treatment including concomitant chemotherapy. While PBT may be less indicated for extremity tumors, exceptions should be made for young patients due to the potential for preserving growth plate cartilage and the lymphatic and vascular–nerve pathways present in the limbs.
- The physical properties of protons allow for a significant escalation in dose, potentially up to approximately 60 Gy Relative Biological Effectiveness (RBE), in the treatment of radioresistant histotypes, such as Malignant Peripheral Nerve Sheath Tumors (MPNSTs) (see Figure 3).
- PBT can have a crucial role in the treatment of pediatric, adolescent and young adult patients with NRSTS associated with genetic syndromes like neurofibromatosis type 1 (NF1), including MPNST, due to the increased risk of radiation-induced carcinogenesis.

## 6. Conclusions

This review paper focuses on the potential role of PBT in the multimodal approach to young patients with NRSTS. Although it remains to be demonstrated that PBT may offer a clear therapeutic superiority compared to other modern photon techniques, PBT should be considered a valid option for patients with NRSTS.

The limited availability of proton therapy facilities currently poses a significant challenge, impeding sufficient data collection. This issue is compounded by the rarity of these tumors and the difficulty in organizing prospective, specific trials. Therefore, there is a pressing need for tailored international cooperation. The pediatric sarcoma and pediatric radiotherapy communities should join efforts and resources to do the following:

- Develop shared guidelines for PBT indications;
- Centralize RT in high-level referral centers: on the one hand, it is advisable that PBT techniques may be developed in the context of clinical studies, by a well-trained multidisciplinary team with experience in managing particle beams; on the other side, centralization is of key value in order to optimize the use of limited resources;
- Improve quality assurance program [63];
- Define international protocols to compare photon and proton radiation techniques in terms of local control and toxicity.

In this context, the recently developed International Soft Tissue SaRcoma ConsorTium (INSTRuCT) could serve as a platform for achieving the aforementioned goals [4,64].

## Figures and Tables

**Table 1:** Articles describing series of pediatric or adolescent patients with NRSTS treated with PBT, focusing on survival outcomes and toxicity. Sex/Age: F (female), M (male); yr (year); Histology: MPNST (Malignant Peripheral Nerve Sheath Tumors), PNST (Peripheral nerve sheath tumors), NRSTS (Non-Rhabdomyosarcoma Soft Tissue Sarcoma); H&N (head and neck); NA (not available); Outcome: OS (overall survival), LC (local control), DC (distant control), LR (local recurrence), RR (regional recurrence), DR (distant recurrence); Toxicity: G3 (grade 3).

Authors (Publication Year)	Type of Study	N° of Patients	Median Age, Sex	Histology	Tumor Site	RT Dose	Aim of RT	Outcome	Toxicity
Janopaul-Naylor J et al. (2021) [46]	Case report	1 pt	13 yr, F	Leiomyosarcoma (high-grade)	Heart	66 GyRBE (surgical bed) 52.8 GyRBE (preoperative tumor extent)	Adjuvant	2-year follow-up: no evidence of disease	Acute Toxicity: no ≥ G3 Late toxicity: mildly prolapsed mitral valve with mild mitral valve regurgitation, intermittent palpitations
Dunn R. et al. (2021) [47]	Case report	1 pt	16 yr, M	MPNST	H&N	NA	Adjuvant	NA	NA
Ye C et al. (2020) [48]	Case report	1 pt	19 yr, M	Synovial sarcoma	Trachea	63 GyRBE (3.5 Gy/fr)	Adjuvant	18 months follow-up: no evidence of disease	NA
Laughlin BS et al. (2023) [49]	Case report	1 pt	17 yr, M	Spindle cell sarcoma (high grade)	Mediastinum	64.8 GyRBE	Definitive	6.5 years: no evidence of disease	Late toxicity: Stage D Class III/IV Constrictive/Restrictive cardiomyopathy, with chronic pericarditis
Bachmann N. et al. (2022) [50]	Retrospective	36 pts	32 yr (3–75) <18 yr (9) 18–39 yr (15) 11 M, 25 F	31 MPNST 5 PNST	- Trunk (20) - Extremities (5) - H&N (11)	64 GyRBE (range, 50–74)	Neoadjuvant (28) Adjuvant (5) Definitive (3) Primary treatment (28) Recurrence (8)	2-year OS, LC, and DC were 75.5%, 73.5%, and 61.2%	Acute toxicity: five G3 (dermatitis, mucositis, and pain) Late Toxicity: four G3 (cataract, osteonecrosis)
Vogel J. et al. (2018) [51]	Retrospective	69 pts with H&N tumors 24 NRSTS	14 yr (1–21) for the 24 NRSTS 15 M, 9 F	NRSTS	H&N	63.0 GyRBE (range 36.0–81.0)	NA	1- and 3-yr OS 93% and 90%. 1- and 3-yr freedom from: - LR: 92% and 85%, - RR: 94% and 86%, - DR: 86% and 78%	Acute toxicity: G3 (oral mucositis, anorexia, dysphagia, dehydration, and dermatitis)
Vazquez M. et al. (2023) [52]	Retrospective	28 pts with H&N tumors, four NRSTS	23.7 yr (15–37.9) 14 M, 14 F	Four NRSTS: three synovial sarcoma, one fibrosarcoma	H&N	63 GyRBE (range 45–74)	NA	5-yr LC, DC and OS were 71.8%, 80.5% and 90.7%	Acute toxicity: 7 G3 (dermatitis, mucositis) Late toxicity: 11 (cataracts, otite media, hearing impairment, sinusitis, osteoradionecrosis, retinopathy)
Hug EB. et al. (2002) [53]	Retrospective	29 pts with skull base tumors, three NRSTS	12 yr (1–19) 14 M, 15 F	3 NRSTS	Skull base	70 GyRBE (range 45–78.6) NRSTS: 50.4 GyRBE (range 50.4–59.6)	Adjuvant	5-yr LC and OS were 72% and 56%	Late toxicity: two motor weakness and sensory deficits
Hwang E. et al. (2023) [54]	Retrospective	495 pts 37 pts adult-type sarcoma	11 yr (0–69) - 348 pts < 16 yrs - 111 pts (16–25 yr)	Non-central nervous system tumor	H&N, abdomen, pelvis, thorax, other	51 GyRBE (range 50.4-55.8)	NA	2-year and 5-year OS for all patients were 88.3% and 82.1%. 2-year and 5-year LC for all patients were 90.3% and 82.9%. LC for adult-type sarcoma 84.4%.	Late toxicity: - 59 G3 (cataracts, musculoskeletal deformity, premature, menopause and hearing impairment) - Seven G4 Three treatment-related secondary malignancy
Million L. et al. (2021) [28]	Sub-analysis of ARST0332 Trial	193 pts 6 pts had PBT	148 pts < 18 yr 45 pts 18–30 yr	Synovial sarcoma (75), MPNST (43), Undifferentiated (30), Other (45)	Body wall, extremity, H&N, visceral	Range 55.8–64.8 GyRBE	Adjuvant, neoadjuvant	NA	NA
